# The prevalence of vegetarians, vegans and other dietary patterns that exclude some animal-source foods in a representative sample of New Zealand adults

**DOI:** 10.1017/S1368980023002677

**Published:** 2023-12-05

**Authors:** James Greenwell, Megan Grant, Leanne Young, Sally Mackay, Kathryn Erica Bradbury

**Affiliations:** 1 Public Health Agency, Manatū Hauora (Ministry of Health), Wellington, New Zealand; 2 Department of Epidemiology and Biostatistics, School of Population Health, University of Auckland, Auckland 1142, New Zealand

**Keywords:** Dietary patterns, Prevalence, New Zealand, Vegetarian, Vegan, Pescatarian, Red meat

## Abstract

**Objective:**

This study aimed to estimate the prevalence of vegetarians, vegans and other dietary patterns that exclude some animal-source foods in New Zealand adults. We also examined socio-demographic and lifestyle correlates of these dietary patterns.

**Design:**

The New Zealand Health Survey is a representative rolling cross-sectional survey of New Zealanders; data from the 2018/19 and 2019/20 waves were used for this analysis. Participants were asked if they completely excluded red meat, poultry, fish/shellfish, eggs or dairy products from their diet.

**Setting:**

New Zealand.

**Participants:**

Adults, aged ≥ 15 years (*n* 23 292).

**Results:**

The prevalence of red-meat excluders (2·89 %), pescatarians (1·40 %), vegetarians (2·04 %) and vegans (0·74 %) was low. After adjustment for socio-demographic and lifestyle factors, women (OR = 1·54, 95 % CI: 1·22, 1·95), Asian people (OR = 2·56, 95 % CI: 1·96, 4·45), people with tertiary education (OR = 1·71, 95 % CI: 1·18, 2·48) and physically active people (OR = 1·36, 95 % CI: 1·04, 1·76) were more likely to be vegetarian/vegan. Those aged ≥ 75 years (OR = 0·28, 95 % CI: 0·14, 0·53) and current smokers (OR = 0·42, 95 % CI: 0·23, 0·76) were less likely to be vegetarian/vegan. Similar associations were seen between socio-demographic and lifestyle factors and the odds of being a red-meat excluder/pescatarian.

**Conclusions:**

Approximately 93 % of New Zealand adults eat red meat and a very small number exclude all animal products from their diets. The Eating and Activity Guidelines for New Zealand adults recommend a plant-based diet with moderate amounts of animal-source foods. A comprehensive national nutrition survey would provide detailed information on the amount of red meat and other animal-source foods that the New Zealand population currently consumes.

There has been increasing interest, amongst scientists and the general public, in the possible health and environmental benefits of vegetarian diets as well as other diets that exclude some animal-source foods. Cross-sectional analyses have shown that vegetarians tend to have lower BMI^(1)^, blood cholesterol concentrations^(2)^ and blood pressure compared with meat-eaters^(1)^. Prospective cohort studies with large numbers of vegetarians have also shown that vegetarians have a lower risk for developing ischaemic heart disease^(3)^, some cancers^(4,5)^ and diabetes^(6,7)^, although may be at higher risk for stroke^(3)^ and hip fracture^(8)^.

Globally, agriculture is responsible for about a quarter of all greenhouse gas emissions^(9)^. In New Zealand (NZ) this estimate is even higher – half of NZ’s total greenhouse gas emissions come from agriculture^(10)^. Generally, the production of animal-source food products results in greater greenhouse gas emissions than plant-source food products (by weight)^(11)^ and therefore substantial reductions in emissions can be achieved through changes to population diets. Recent research from NZ has modelled shifting the adult population from the current NZ diet to a vegetarian diet, and the results indicate that this would reduce dietary-related greenhouse gas emissions by about a quarter^(12)^.

There are various methods for assessing vegetarian, vegan and other non-meat dietary patterns. Market research companies have estimated the proportion of New Zealanders that are almost or always vegetarian or vegan; however, the reliability of these estimates is uncertain. In one survey (*n* 5983) conducted in 2014/2015, 10·3 % of New Zealanders aged 14 years and over said the food they eat is all or almost all vegetarian^(13)^. In another survey by a different company (*n* 1517), conducted in 2021, 19 % of participants reported always or mostly maintaining a vegetarian or vegan diet^(14)^. The specific sampling methods used in these market research polls are not clear. In addition, the questions used in the surveys ask people to self-identify as vegetarian/vegan or mostly vegetarian/vegan, which will overestimate prevalence compared with asking people about the food groups that they consume or exclude^(15)^.

The New Zealand Health Survey (NZHS) is a rolling survey, administered annually to a representative sample of NZ adults. In the 2018/19 and the 2019/20 surveys, the participants were asked whether they exclude red meat, poultry, fish or seafood or dairy products from their diet. The objective of this study is to quantify the prevalence of vegetarian, vegan and other dietary patterns that exclude some animal-source foods in a large representative sample of the NZ adult population and to examine the associations between socio-demographic, lifestyle and physiological characteristics and these dietary patterns.

## Methods

### Study population and survey design

The NZHS is an important cross-sectional survey administered annually that aims to monitor the health of the NZ population. The main objectives of the sampling design are to provide a range of prevalence estimates for health behaviours and conditions by age, ethnicity and geographical region^(16)^. This study uses data from the 2018/19 and 2019/20 waves of data collection. The 2018/19 sample was selected from 1 July 2018 to 30 June 2019^(17)^. The 2019/20 sample was selected from July 1 2019 and was supposed to run until June 2020 but was suspended on March 19 2020 due to the government restrictions put in place in response to the COVID-19 pandemic; thus, the sample size and response rate for the 2019/20 NZHS are slightly lower^(16)^.

The sampling strategy and methodology of the NZHS have been described previously^(16–18)^. Briefly, the NZHS has a multi-stage, stratified, probability-proportional-to-size sampling design. The target population for the NZHS is the usually resident population of NZ. Two sampling frames are used: participants are selected from an area-based sample and a list-based electoral roll sample. For the area-based sampling frame, first a sample of Statistics New Zealand’s household survey frame primary sampling units are selected, second, a random sample of households from within each primary sampling unit are selected and third, one adult (aged 15 years or over) and one child (14 years or younger) are selected at random from within each household. The electoral roll sampling frame is used to increase the sample size of Māori participants and generally follows the same approach as the area-based sampling frame, but at the second stage households within the primary sampling unit are selected from the list of households where any person has self-identified on the electoral roll as having Māori ancestry.

Data collection for the NZHS takes place in the participants’ homes. Trained interviewers enter participant responses directly into a laptop. In addition, participants complete some sections of the interview by themselves using the laptop. The interviews gather information on socio-demographics as well as a variety of health-related topics including long-term conditions; health status and health behaviours and risk factors. In 2018/19 and 2019/20, a module on dietary habits was included. In addition to the interview, height and weight and blood pressure are measured following standardised protocols.

## Classification of dietary patterns

Diet pattern categorisation in this study follows the exclusion criteria method used by Valdes *et al.* ^(19)^. A single dietary exclusion question was included in the dietary habits questionnaire module and was used to classify participants into dietary pattern groups. The question asked: ‘Do you completely exclude any of the following food groups from your diet?’ The response options were ‘Red meat (e.g. beef, pork, mutton, lamb, goat and venison); chicken or poultry (e.g. turkey and duck); fish or other seafood; eggs; dairy products (e.g. milk and cheese); gluten sources (e.g. wheat and barley); nuts’. Participants were also told that ‘Completely exclude means you never eat it on its own, or as part of a prepared dish’. Survey respondents had the option to choose multiple categories based on what they completely exclude. For the purposes of this study, the responses for excluding gluten sources and nuts were not used.

Food exclusion categories were coded into one of four prioritised (and mutually exclusive) categories and those not reporting exclusions were categorised as red-meat eaters. Table 1 provides a summary of the dietary pattern classifications. Red-meat excluders were defined as excluders of red meat, pescatarians were defined as excluders of red meat and poultry, vegetarians were defined as those who excluded red meat, poultry, and fish/seafood, and vegans were defined as excluders of red-meat, poultry, fish/seafood, eggs, and dairy products. Those reporting none of the above exclusions were categorised as red-meat eaters.

**Table 1:** Dietary pattern definitions and weighted adult prevalence from the New Zealand Health Survey 2018/19 and 2019/20

						Prevalence					
	Reported dietary exclusions*					Men (*n* 9804)		Women (*n* 13 488)		All (*n* 23 292)	
Dietary pattern	Red meat	Poultry	Fish/shellfish	Eggs	Dairy	%	95 % CI	%	95 % CI	%	95 % CI
Red-meat eater						94·97	94·3, 95·58	90·95	90·04, 91·80	92·93	92·33, 93·53
Red-meat excluder	X					2·05	1·65, 2·52	3·70	3·24, 4·20	2·89	2·56, 3·22
Pescatarian	X	X				0·69	0·49, 0·95	2·08	1·68, 2·55	1·40	1·16, 1·64
Vegetarian	X	X	X			1·64	1·31, 1·97	2·43	2·00, 2·86	2·04	1·77, 2·32
Vegan	X	X	X	X	X	0·65	0·43, 0·87	0·83	0·63, 1·04	0·74	0·57, 0·91

* Participants were asked ‘Do you completely exclude any of the following food groups from your diet?’ The response options were ‘red meat (e.g. beef, pork, mutton, lamb, goat and venison); chicken or poultry (e.g. turkey, duck); fish or other seafood; eggs; dairy products (e.g. milk, cheese)’. Participants were also told that ‘completely exclude means you never eat it on its own, or as part of a prepared dish’.

### Covariates

Socio-economic and lifestyle variables used in this study were derived according to conventional NZHS methods^(16)^. They include sex (men, women), age (15–24, 25–44, 45–64, 65–74 and ≥ 75 years), total response ethnicity (Māori, Pacific, Asian and NZ European/Other ethnicities (including Middle Eastern, Latin American, African and other ethnicities); total response ethnicity allows for participants to be assigned to more than one ethnicity), education level (less than upper secondary, upper secondary and tertiary/other), the area-based 2013 New Zealand Deprivation Index (in quintiles from least deprived areas to most deprived areas)^(20)^, BMI (continuous, kg/m^2^), systolic blood pressure (continuous using the mean of the second and third measurements, mm Hg), smoking status (not current, current) and physical activity (active, inactive; participants are classified as active if ≥ 150 min of time spent on physical activity in the past 7 d and ≥ 30 min of moderate-intensity physical activity on ≥ 5 of the past 7 d).

### Statistical analysis

The analysis of the NZHS data was carried out in R3·6·3 using the *nzhealthsurvey version 0·0·4* package developed by Steven Johnston for the NZ Ministry of Health. Survey weights were used in all analyses so that estimates of prevalence and means are representative of the usually resident adult population of NZ. The survey uses the calibrated weighting method to construct survey weights that rate up the responding sample to represent the target population. This method considers the probability of selection of each respondent and uses external population benchmarks (typically based on the most recent population census) to correct for any discrepancies between the sample and population benchmarks (by age, sex, ethnicity and the 2013 New Zealand Index of Deprivation).

Missing data comprised < 1 % of total sample (159 responses) and was dropped following an examination indicating data was missing completely at random. Descriptive statistics are provided for each variable using the entire sample excluding observations where dietary habits questionnaire responses were dropped. The analysis of BMI and blood pressure used slightly smaller samples.

Weighted population prevalence estimates with 95 % confidence intervals were calculated for each of the four dietary patterns, overall and by sex. Due to small samples, particularly for pescatarians and vegans, for all other analyses the red-meat excluders were combined with pescatarians and the vegetarians were combined with vegans. We conducted bivariate analyses of the dietary patterns and the social and demographic variables using Rao-Scott *χ* ^2^ tests for the categorical variables and linear regression models for the continuous variables (e.g. age, BMI and systolic blood pressure, which were treated as dependent variables). Multinomial logistic regression was used to calculate adjusted OR with 95 % CI. The models were mutually adjusted for sex, age group, total response ethnicity, education category, NZDep2013 quintile, BMI, systolic blood pressure, physical activity categories and smoking status.

## Results

The response rate for the adult portion of the NZHS was 80 % in 2018/19 and 75 % in 2019/20. The sample size for this study includes 23 292 who answered the dietary exclusion question in these two waves of data collection. A sub-sample of participants (*n* 21 481) had BMI and blood pressure measurements.

Table 1 presents the population weighted prevalence estimates for each dietary patterns overall and by sex. Most adults reporting some form of animal-source food exclusion are red-meat excluders (2·89 %) followed by vegetarians (2·04 %), pescatarians (1·40 %) and vegans (0·74 %). The vast majority of the adult population (92·9 %) of NZ are red-meat eaters.

Table 2 and Table 3 present the unadjusted prevalence of the dietary patterns of interest by participant characteristics for men and women, respectively. Among men and women, vegetarians/vegans were younger (mean age for men = 36·21 years, for women = 38·72 years) than red-meat eaters (mean age for men = 45·23 years, for women = 46·73 years). For both men and women, there was a much higher prevalence of red-meat excluders/pescatarians among Pacific people (7·26 % for men, and 8·15 % for women) compared with NZ Europeans (1·37 % for men and 4·00 % for women). There was a much higher prevalence of vegetarians/vegans among Asian people (6·42 % for men and 8·53 % for women) compared with NZ Europeans (1·67 % for men and 2·82 % for women). For both men and women, among those with a tertiary education, there was a higher prevalence of red-meat excluders/pescatarians (3·96 % for men and 6·62 % for women) compared with those with less than upper secondary schooling (1·79 % for men and 4·37 % for women). There was also a higher prevalence of vegetarians/vegans (3·13 % for men and 4·35 % for women) in those with tertiary education compared with those with less than upper secondary schooling (1·39 % for men and 1·88 % for women). Among men and women the mean BMI was lower among red-meat excluders/pescatarians (26·80 kg/m^2^ in men and 26·69 kg/m^2^ in women) and vegetarians/vegans (25·73 kg/m^2^ in men and 26·12 kg/m^2^ in women), compared with red-meat eaters (28·25 kg/m^2^ in men and 28·29 kg/m^2^ in women). Mean systolic blood pressure was lower in vegetarians/vegans (124·80 mmHg) compared with red-meat eaters (128·56 mmHg) among men and lower in red-meat eaters/pescatarians (118·77 mmHg) and vegetarians/vegans (115·21 mmHg) compared with red-meat eaters (121·66 mmHg) among women. For both men and women, there was a lower prevalence of vegetarians/vegans in current smokers (0·86 % in men and 1·26 % in women) compared with not current smokers (2·55 % in men and 3·51 % in women).

**Table 2:** Unadjusted demographic and biometric characteristics by dietary pattern in men*

	Dietary pattern						
	Red-meat eater		Red-meat excluder/Pescatarian		Vegetarian or vegan		
Characteristic	%	95 % CI	%	95 % CI	%	95 % CI	*P* value
Total							
Age (years)							
Mean	45·23	45·07, 45·40	43·10	40·50, 45·70	36·21	32·78, 39·65	<0·001
15–24	94·60	92·74, 96·11	2·05	1·20, 3·27	3·34	2·12, 5·00	<0·001
25–44	93·32	91·90, 94·57	3·62	2·65, 4·80	3·06	2·35, 3·91	
45–64	95·97	95·06, 96·75	2·48	1·89, 3·20	1·55	1·07, 2·18	
65–74	96·72	95·35, 97·77	2·19	1·31, 3·42	1·10	0·53, 2·01	
75+	96·78	95·40, 97·84	2·21	1·35, 3·41	1·01	0·39, 2·12	
Total response ethnicity							
NZ European/other	96·33	95·62, 96·96	1·37	0·69, 2·43	1·67	1·23, 2·21	
Māori	97·57	96·35, 98·47	2·52	1·02, 5·12	1·06	0·50, 1·96	<0·001
Pacific	96·36	93·50, 98·20	7·26	5·34, 9·59	1·12	0·26, 3·11	
Asian	86·33	83·60, 88·75	2·00	1·58, 2·49	6·42	4·91, 8·22	
Education							
Less than upper secondary	96·82	95·72, 97·70	1·79	1·12, 2·70	1·39	0·77, 2·30	<0·001
Upper secondary	96·89	96·05, 97·60	1·45	1·02, 1·99	1·66	1·08, 2·44	
Tertiary/other	92·91	91·64, 94·04	3·96	3·07, 5·01	3·13	2·51, 3·86	
NZDep2013 quintile†							
1	96·14	94·84, 97·19	1·99	1·29, 2·92	1·87	1·11, 2·95	0·128
2	94·07	92·35, 95·50	3·26	2·18, 4·68	2·67	1·81, 3·78	
3	96·27	95·11, 97·22	2·02	1·32, 2·96	1·71	1·03, 2·67	
4	93·87	92·05, 95·38	3·54	2·31, 5·17	2·59	1·80, 3·60	
5	94·46	92·85, 95·80	2·94	1·95, 4·24	2·60	1·77, 3·68	
BMI	28·25	28·09, 28·40	26·80	26·02, 27·58	25·73	24·95, 26·51	<0·001
Systolic blood pressure (mm Hg)	128·56	128·04, 129·07	126·50	124·28, 128·72	124·80	121·97, 127·62	0·004
Smoking status							
Not current	94·57	93·83, 95·24	2·89	2·38, 3·47	2·55	2·12, 3·04	0·001
Current	97·15	95·91, 98·10	1·98	1·22, 3·03	0·86	0·38, 1·68	
Physical activity‡							
Inactive	95·31	94·39, 96·12	2·55	1·95, 3·27	2·14	1·63, 2·75	
Active	94·74	93·86, 95·53	2·91	2·29, 3·63	2·35	1·82, 2·99	0·612

* There are 9804 men in the sample, except for the BMI and SBP measures for which there is information available on 9237 men.

† NZDep2013 is the area-based 2013 New Zealand Deprivation Index in quintiles from least deprived (quintile 1) to most deprived (quintile 5).

‡ Participants are considered physically active if time spent on physical activity in the past 7 days is at least 150 min, and they have done 30 min or more of moderate-intensity physical activity per day on at least five of the past 7 days.

**Table 3:** Unadjusted demographic and biometric characteristics by dietary pattern in women*

	Dietary pattern						
	Red-meat eater		Pescatarian/Red-meat excluder		Vegetarian or vegan		
Characteristic	%	95 % CI	%	95 % CI	%	95 % CI	*P* value
Total							
Age (years)							
Mean	46·73	46·45, 47·01	44·01	42·17, 45·85	38·72	35·04, 42·41	<0·001
15–24	89·16	86·42, 91·52	6·73	4·98, 8·87	4·10	2·87, 5·66	<0·001
25–44	89·05	87·43, 90·53	6·17	5·11, 7·38	4·77	3·84, 5·85	
45–64	91·83	90·44, 93·07	5·49	4·49, 6·64	2·68	1·90, 3·68	
65–74	93·28	91·46, 94·82	5·46	4·08, 7·13	1·26	0·64, 2·22	
75+	95·21	93·85, 96·35	4·07	3·13, 5·20	0·71	0·28, 1·48	
Total response ethnicity							
NZ European/other	91·39	90·25, 92·43	4·00	3·07, 5·12	2·82	2·30, 3·42	
Māori	94·23	92·80, 95·45	4·97	3·12, 7·45	1·77	1·06, 2·76	<0·001
Pacific	94·16	91·43, 96·23	8·15	6·45, 10·12	0·88	0·15, 2·73	
Asian	83·32	80·28, 86·07	5·79	4·99, 6·68	8·53	6·66, 10·73	
Education							
Less than upper secondary	93·75	92·59, 94·77	4·37	3·52, 5·36	1·88	1·21, 2·79	<0·001
Upper secondary	92·18	90·36, 93·75	5·42	4·12, 6·98	2·41	1·78, 3·17	
Tertiary/other	89·03	87·86, 90·13	6·62	5·80, 7·52	4·35	3·67, 5·11	
NZ Dep2013 quintile†							
1	91·47	89·24, 93·38	6·78	5·14, 8·75	1·75	1·06, 2·71	0·003
2	90·21	88·19, 91·99	6·02	4·62, 7·70	3·77	2·73, 5·06	
3	89·32	86·62, 91·64	6·00	4·46, 7·86	4·68	3·48, 6·15	
4	91·46	89·90, 92·86	5·11	4·23, 6·12	3·42	2·49, 4·59	
5	92·34	90·83, 93·67	5·01	4·12, 6·04	2·65	1·89, 3·60	
BMI	28·29	28·12, 28·46	26·69	26·01, 27·37	26·12	25·40, 26·83	<0·001
Systolic blood pressure (mm Hg)	121·66	121·22, 122·10	118·77	116·89, 120·66	115·21	113·13, 117·29	<0·001
Smoking status							
Not current	90·31	89·30, 91·25	6·18	5·47, 6·95	3·51	3·03, 4·05	<0·001
Current	95·57	93·92, 96·88	3·18	2·27, 4·31	1·26	0·54, 2·47	
Physical activity‡							
Inactive	92·00	90·88, 93·03	5·12	4·34, 6·00	2·87	2·31, 3·53	0·018
Active	89·77	88·37, 91·05	6·50	5·59, 7·52	3·73	2·96, 4·63	

* There are 13 488 women in the sample, except for the BMI and SBP measures for which there is information available on 11 971 women.

† NZDep2013 is the area-based 2013 New Zealand Deprivation Index in quintiles from least deprived (quintile 1) to most deprived (quintile 5).

‡ Participants are considered physically active if time spent on physical activity in the past 7 days is at least 150 min and they have done 30 min or more of moderate-intensity physical activity per day on at least five of the past 7 days.

Table 4 presents the adjusted odds of excluding red meat/being pescatarian or being vegetarian/vegan compared with being a red-meat eater, after mutually adjusting for sex, age group, total response ethnicity, education category, NZDep2013, BMI, systolic blood pressure, smoking status and physical activity status. After adjusting for these other factors, women were more likely to be red-meat excluders/pescatarians (OR = 2·32, 95 % CI: 1·87, 2·89, *P* < 0·001) and vegetarians/vegans (OR = 1·55, 95 % CI: 1·22, 1·95, *P* < 0·001) compared with men. Those aged 75 years and older were less likely to be red-meat excluders/pescatarians (OR = 0·68, 95 % CI: 0·47, 0·98, *P* = 0·040) and vegans/vegetarians (OR = 0·27, 95 % CI: 0·14, 0·53, *P* < 0·001) and those aged 65–74 years were also less likely to be vegetarian/vegan (OR = 0·39, 95 % CI: 0·22, 0·71, *P* = 0·002), compared with those aged 15–24 years. Asian people were more likely to be red-meat excluders/pescatarians (OR = 1·80, 95 % CI: 1·40, 2·31, *P* < 0·001) and vegetarians/vegans (OR = 2·55, 95 % CI: 1·95, 3·35, *P* < 0·001), compared with NZ Europeans/others. Those with tertiary education were more likely to be red-meat excluders/pescatarians (OR = 1·53, 95 % CI: 1·19, 1·96, *P* = 0·001) and vegetarians/vegans (OR = 1·70, 95 % CI: 1·17, 2·46, *P* = 0·005) compared with those with less than upper secondary education. Those with higher BMI were less likely to be red-meat excluders/pescatarians (OR for every 1 unit increase in BMI = 0·97, 95 % CI: 0·95, 0·99, *P* = 0·001) and vegetarians/vegans (OR for every 1 unit increase in BMI = 0·96, 95 % CI: 0·94, 0·98, *P* < 0·001). After adjustment for other factors, there was no association between dietary patterns and systolic blood pressure. Current smokers were less likely to be red-meat excluders/pescatarians (OR = 0·62, 95 % CI: 0·43, 0·89, *P* = 0·009) and vegetarians/vegans (OR = 0·43, 95 % CI: 0·24, 0·77, *P* = 0·005) compared to those that were not current smokers. Finally, those who were active were more likely to be red-meat excluders/pescatarians (OR = 1·30, 95 % CI: 1·08, 1·56, *P* = 0·005) and vegetarians/vegans (OR = 1·34, 95 % CI: 1·03, 1·75, *P* = 0·027) compared with those that were inactive.

**Table 4:** Adjusted odds of excluding red meat/being pescatarian or being vegetarian/vegan compared with being a red-meat eater among a nationally representative sample of NZ adults 15+

	Red-meat excluders/pescatarians odds			Vegetarian/vegan odds		
	OR	95 % CI	*P* value	OR	95 % CI	*P* value
Sex						
Men	Reference		<0·001	Reference		<0·001
Women	2·32	1·87, 2·89		1·55	1·22, 1·95	
Age (years)						
15–24	Reference			Reference		
25–44	0·95	0·71, 1·27	0·727	0·94	0·67, 1·33	0·737
45–64	0·87	0·64, 1·19	0·389	0·59	0·39, 0·89	0·012
65–74	0·86	0·57, 1·29	0·458	0·39	0·22, 0·71	0·002
75+	0·68	0·47, 0·98	0·040	0·27	0·14, 0·53	<0·001
Total response ethnicity						
NZ European/other	Reference			Reference		
Māori	0·79	0·56, 1·10	0·163	0·65	0·42, 1·01	0·060
Pacific	1·25	0·79, 1·96	0·340	0·44	0·18, 1·07	0·069
Asian	1·80	1·40, 2·31	<0·001	2·55	1·95, 3·35	<0·001
Education						
Less than upper secondary	Reference			Reference		
Upper secondary	1·01	0·73, 1·39	0·969	1·02	0·65, 1·59	0·936
Tertiary/other	1·53	1·19, 1·96	0·001	1·70	1·17, 2·46	0·005
NZDep2013 quintile*						
Least deprived	Reference			Reference		
2	1·07	0·78, 1·47	0·683	1·92	1·28, 2·89	0·002
3	1·00	0·71, 1·41	1·000	1·82	1·15, 2·88	0·010
4	1·14	0·87, 1·49	0·352	1·93	1·28, 2·91	0·002
5	1·17	0·83, 1·63	0·366	2·23	1·44, 3·45	0·000
BMI						
Per 1 kg/m^2^	0·97	0·95, 0·99	0·001	0·96	0·94, 0·98	<0·001
Systolic blood pressure						
Per 1 mmHg	1·00	0·99, 1·01	0·822	1·00	0·99, 1·01	0·598
Smoking status						
Not current	Reference			Reference		
Current	0·62	0·43, 0·89	0·009	0·43	0·24, 0·77	0·005
Physically active†						
Inactive	Reference			Reference		
Active	1·30	1·08, 1·56	0·005	1·34	1·03, 1·75	0·027

* NZDep2013 is the area-based 2013 New Zealand Deprivation Index in quintiles from least deprived (quintile 1) to most deprived (quintile 5).

† Participants are considered physically active if time spent on physical activity in the past 7 days is at least 150 min, and they have done 30 min or more of moderate-intensity physical activity per day on at least five of the past 7 days.

## Discussion

In this large, recent representative sample of adult New Zealanders, the prevalence of dietary patterns that exclude animal-source foods was low, with only 2·89 % of the sample classified as red-meat excluders, 1·40 % as pescatarians, 2·04 % as vegetarians and 0·74 % as vegans. After adjusting for socio-demographic and lifestyle factors, women, younger people, Asian people, more educated people, people with lower BMI, people who currently do not smoke and physically active people were more likely to be red-meat excluders/pescatarians and vegetarians/vegans.

In the 2008/09 NZ Adult Nutrition Survey, 94·5 % of the participants reported eating red meat in the previous 4 weeks^(21)^, which is in line with the up-to-date estimate from the current study, where 93 % of participants reported eating red meat, indicating that the prevalence of red-meat eaters has probably remained fairly stable over the past 15 years. The prevalence of vegetarians and vegans (< 3 % combined) found in the current study is much lower than that found in two fairly recent market research polls. The research polls conducted in 2014/15 and 2019 reported that 10·3 % of New Zealanders aged 14 years and over said the food they eat is all or almost all vegetarian,^(13)^ and 19 % of participants reported always or mostly maintaining a vegetarian or vegan diet^(14)^, respectively. The sample we used is much larger (*n* 23 292), and representative of the NZ resident population and may avoid other potential biases of consumer polls. Another key difference between our results and the market research polls that would partly explain our much lower prevalence is that participants in the NZHS were asked if they completely exclude meat, poultry, fish, dairy and eggs from their diet. Whereas participants in the market research polls were asked if almost all the food eaten is vegetarian or if they mostly maintained a vegetarian or vegan diet, and what people interpret as ‘almost’ or ‘mostly’ a vegetarian diet is subjective and could include a range of meat intakes. The 2018 wave of the NZ Values and Attitudes Study, where participants are mainly randomly sampled from the electoral roll with oversampling of some regions and ethnicities, included an open-ended question about dietary habits. Participants were asked: ‘How would you describe your dietary behaviour? (e.g. meat and veges, vegetarians, vegan, halal, pescatarian, etc.)’, with researchers coding different dietary patterns from the participants’ responses^(22)^. Despite the different approach to dietary pattern classification, the study found only slightly higher prevalence of dietary patterns to our sample, with 4·5 % classified as vegetarians and 1·1 % classified as vegan. Also similar to our study, in the NZ Values and Attitude study, women were more likely to be vegetarians than men^(22)^.

In the 2015 Canadian Community Health Survey (*n* 20 477), a very similar question about dietary exclusions was asked, and Valdes et al.^(19)^ used the same categorisation we used to define dietary patterns (although they refer to red-meat eaters as ‘non-plant based’). In the Canadian survey, 2·8 % of participants were red-meat excluders, 0·7 % pescatarians, 1·3 % vegetarian and 0·3 % vegan^(19)^. As well as having similar prevalence of these dietary patterns to our survey, the Canadian survey also found that women were more likely to be red-meat excluders/pescatarians than men and that South Asians were much more likely to be red-meat excluders/pescatarians and vegetarians/vegans than White participants. Cultural and religious differences in traditional cooking ingredients and recipes are likely to drive these ethnic differences. In addition, those with a Bachelor’s degree or higher were more likely to be red-meat excluders/pescatarians and vegetarians/vegans than those with only high-school equivalent education^(19)^.

In our study, there was no clear association between our measure of area-based deprivation and dietary patterns that exclude animal-source foods. Similarly, in the 2015 Canadian Community Health Survey, household income did not show a clear association with dietary patterns^(19)^. However, in both surveys, those with higher education were more likely to be red-meat excluders/pescatarians and vegetarians/vegans. There may be several factors at play that explain why no clear association is seen with area-based deprivation or household income. It is possible that although those who are highly educated are more likely to exclude animal products from the diet, the lifestyles, preferences and types of occupations of highly educated red-meat excluders, pescatarians, vegetarians and vegans may differ from comparably highly educated red-meat eaters. It is also possible that the price of meat and other animal-source foods is prohibitive to those on very low incomes. We were unable to determine whether people with food insecurity were more likely to exclude animal-source foods because questions on food security were only included in the 2019/20 wave of the NZHS. In our unadjusted analysis, there were significant differences in systolic blood pressure across dietary groups; vegetarians/vegans had lower systolic blood pressure compared with red-meat eaters. However, this difference disappeared after adjustment, indicating that it was explained by the other factors, including BMI.

The NZHS is a large and representative survey, and the analyses uses weighting so that estimates of prevalence and means are representative of the usually resident adult population of NZ. We used strict definitions to classify participants as red-meat excluders, pescatarians, vegetarians or vegans. It is possible that some participants did not consider processed meat when they were asked if they excluded red meat from their diet, because in earlier questions that asked about the frequency of consumption of main food groups, processed meat was separated out from red meat^(23)^. We think that this would have only affected the responses of participants who excluded unprocessed red meat but ate processed meat – some of these participants may have mistakenly said that they exclude red meat, when in fact they ate processed meat. However, we think that the proportion of participants who excluded unprocessed red meat but ate processed meat would be very small, and therefore unlikely to have had a major influence on our estimate of the prevalence of red-meat excluders. As we only had information on whether people completely excluded specific animal-source foods from the diet, we were unable to determine if some people who were classified as red-meat eaters ate low amounts of red meat and could be considered plant-based or flexitarian; however, these dietary patterns are not well defined. The NZHS does not include a full dietary assessment, and the most recent Adult Nutrition Survey was carried out in 2008/09. A new comprehensive national nutrition survey would make it possible to quantify the amount of meat and other animal-source foods consumed by the population and allow for a more nuanced exploration of those eating small amounts of meat. A comprehensive nutrition survey would also enable researchers to explore the extent to which the diet of the NZ population aligns with the Eating and Activity guidelines for New Zealand Adults, which recommend a largely plant-based diet that contains moderate amounts of animal-source foods^(24)^ and the EAT-LANCET diet, which is designed for optimal planetary and human health and similarly allows small amounts of red meat (up to 196 g/week) and other animal-source foods^(25)^.

We found that people of Asian ethnicity were more likely to be red-meat excluders/ pescatarian and vegetarian/vegan. People of Asian ethnicity living in NZ are heterogenous, and previous studies in NZ have shown that South Asians are more likely to avoid animal products compared with East and South-East Asians^(26)^. Unfortunately, the sampling frame of the NZHS constrains our ability to publish disaggregated statistics for participants of different Asian ethnicities, and therefore we are unable to investigate differences in the prevalence of dietary patterns between different Asian ethnicities.

The 2019/20 wave of data collection for the NZHS was terminated slightly prematurely due to government restrictions put in place in response to the COVID-19 pandemic, although the final sample used in this study consisted of over 20 000 adults. This analysis provides a baseline which could be compared with future waves of the NZHS that include a dietary habits questionnaire. It would be interesting to see if the COVID-19 pandemic and/or rising inflation^(27)^ influences the net prevalence of vegetarians and other diets that exclude some animal-source foods.

In conclusion, this study provides a timely analysis of dietary exclusions in a representative sample of over 20 000 NZ adults. Our results indicate that the prevalence of red-meat excluders, pescatarians, vegetarians, and vegans was low, and approximately 93 % of New Zealanders eat red meat. Women, Asian people, highly educated people, people with lower BMI, physically active people and people who do not currently smoke were more likely to be red-meat excluders/pescatarians and vegetarians/vegans. It would be of great interest to include the dietary habits module in future NZHS to be able to determine trends in the prevalence of dietary patterns that exclude some animal-source foods. A national nutrition survey will enable the quantification of the amount of animal and plant-source foods consumed by the NZ population and the alignment of the NZ diet to national and global dietary guidelines, which recommend a largely plant-based diet with small amounts of animal-source foods for human and planetary health.
